# Integrin linked kinase (ILK) regulates podosome maturation and stability in dendritic cells^?^

**DOI:** 10.1016/j.biocel.2014.01.021

**Published:** 2014-05

**Authors:** Mercedes Griera, Ester Martin-Villar, Inmaculada Banon-Rodríguez, Michael P. Blundell, Gareth E. Jones, Ines M. Anton, Adrian J. Thrasher, Manuel Rodriguez-Puyol, Yolanda Calle

**Affiliations:** a Department of Physiology, Facultad de Medicina, Universidad de Alcalá, Campus Universitario s/n, Alcalá de Henares, Madrid 28871, Spain; b Cancer Biology Department, Instituto de Investigaciones Biomédicas “Alberto Sols”, CSIC-UAM, Madrid 28029, Spain; c Cellular and Molecular Department, Centro Nacional de Biotecnología (CNB-CSIC), Madrid 28049, Spain; d Wolfson Centre for Gene Therapy, Molecular Immunology Unit, Institute of Child Health, University College London, WC1N UK; e Randall Division of Cell & Molecular Biophysics, King's College London, London SE1 1UL, UK; f Department of Haemato-oncology, King's College London, London SE5 9NU, UK

**Keywords:** ILK, Dendritic cell, Podosome, WASP, PI3K

## Abstract

Podosomes are integrin-based adhesions fundamental for stabilisation of the leading lamellae in migrating dendritic cells (DCs) and for extracellular matrix (ECM) degradation. We have previously shown that soluble factors and chemokines such as SDF 1-a trigger podosome initiation whereas integrin ligands promote podosome maturation and stability in DCs. The exact intracellular signalling pathways that regulate the sequential organisation of podosomal components in response to extracellular cues remain largely undetermined. The Wiskott Aldrich Syndrome Protein (WASP) mediates actin polymerisation and the initial recruitment of integrins and associated proteins in a circular configuration surrounding the core of filamentous actin (F-actin) during podosome initiation. We have now identified integrin linked kinase (ILK) surrounding the podosomal actin core. We report that DC polarisation in response to chemokines and the assembly of actin cores during podosome initiation require PI3K-dependent clustering of the Wiskott Aldrich Syndrome Protein (WASP) in puncta independently of ILK. ILK is essential for the clustering of integrins and associated proteins leading to podosome maturation and stability that are required for degradation of the subjacent extracellular matrix and the invasive motility of DCs across connective tissue barriers.

We conclude that WASP regulates DCs polarisation for migration and initiation of actin polymerisation downstream of PI3K in nascent podosomes. Subsequently, ILK mediates the accumulation of integrin-associated proteins during podosome maturation and stability for efficient degradation of the subjacent ECM during the invasive migration of DCs.

## 1 Introduction

The directed migration of dendritic cells (DCs) through peripheral tissues is crucial for their function as T and B cell activators during the immune response and failure of appropriate migration can result in immunodeficiency, autoimmune responses or chronic inflammation (Angeli and Randolph, 2006). Many of the chemokines regulating DC chemotaxis are known but there is an incomplete understanding of the regulation of the cytoskeletal and adhesion remodelling that drives DC motility. We and others have shown an absolute requirement of integrin and actin-based adhesive structures called podosomes for normal migration and chemotactic responses of immature DCs (Calle et al., 2006a, 2008; Linder, 2009). Podosomes are highly dynamic adhesions involved in migration of cells that have to cross and invade boundaries (Calle et al., 2006a, 2008). They are characterised by a distinctive organisation, consisting of a core of F-actin surrounded by a circular array of integrins and integrin associated proteins (Calle et al., 2006a, 2008; Linder, 2009). We have previously shown that chemotactic factors such as SDF 1-a trigger podosome initiation whereas integrin ligands including fibronectin and ICAM-1 promote podosome maturation and stability behind the leading edge of motile DCs (Chou et al., 2006; Monypenny et al., 2011). In the absence of stimulation with chemotactic factors DCs remain stationary and attach on integrin ligands through focal contacts (Monypenny et al., 2011). The intracellular signalling that allows the remodelling of adhesions from focal contacts to podosomes leading to the transition from a stationary to a motile phenotype in DCs remains largely unknown (Linder, 2009; Murphy and Courtneidge, 2011).

The first step for podosome initiation involves a burst of actin polymerisation leading to the assembly of a conical core of F-actin (Luxenburg et al., 2012). This is followed by the initial organisation of integrins and integrin-associated proteins in a circular array surrounding the core of F-actin (Monypenny et al., 2011). Subsequent binding of integrins to their ligands increases podosome size by further accumulation of integrins and F-actin leading to podosome maturation and increased adhesion stability. Failure to accumulate integrins and associated proteins forming a ring around in the nascent actin cores of podosomes results in rapid podosome turnover (Macpherson et al., 2012) and abnormal degradation of the subjacent extracellular matrix (Banon-Rodriguez et al., 2011; Dovas et al., 2009).

The Wiskott Aldrich Syndrome Protein (WASP) and the WASP Interacting Protein (WIP) comprise a functional unit that regulates actin polymerisation and integrin remodelling leading to polarisation and podosome initiation in DCs (Calle et al., 2008; Chou et al., 2006; Monypenny et al., 2011) and other myeloid cells (Calle et al., 2008; Cammer et al., 2009; Chabadel et al., 2007; Jones et al., 2002; Linder, 2009). Mutations in the gene coding for WASP result in abnormal adhesion and cytoskeletal organisation of leukocytes (including lack of podosomes in myeloid cells) that largely contribute to the phenotype of clinical diseases including the Wiskott Aldrich Syndrome (WAS) and X-linked thrombocytopenia (Calle et al., 2008). In the absence of WASP or WIP DCs fail to polarise and adhere to integrin ligands by assembling focal contacts, which are unable to degrade the subjacent extracellular matrix (Banon-Rodriguez et al., 2011; Chou et al., 2006; Monypenny et al., 2011). The exact signalling pathways during podosome maturation that may sustain clustering of integrin and integrin-associated proteins around the nascent F-actin core driven by WASP/WIP in podosomes has not yet been determined.

In the present study we show that integrin linked kinase (ILK) is required for the accumulation of integrin-associated proteins in podosome rings downstream of WASP-mediated initiation of the actin core in podosomes. We report that ILK plays a key role in the regulation of the adhesive properties and the invasive motility of DCs across extracellular matrix-based barriers.

## 2 Materials and methods

### 2.1 Animals

Conditional inactivation of the ILK gene was accomplished as previously described (Herranz et al., 2012) by crossing mice carrying the floxed ILK allele (genotype: ILKfl/fl) with CreERT transgenic mice, which express Cre under the control of the cytomegalovirus promoter. Eight-week-old male mice were injected intraperitoneally with a tamoxifen solution once a day for 5 consecutive days. Animals were killed 20 days after the last injection. All animal procedures were approved by the institutional animal care and use committee from the University of Alcala, Madrid (Spain). The mouse colonies were established from mice obatined from S. Shoukat, Department of Integrative Oncology, BC Cancer Research Center, Vancouver, BC, Canada. Pathogen free C57Bl/6 mice purchased from Harlan and WASP-null mice on a C57Bl/6 background were bred in our own animal facility in pathogen free conditions. All animals were handled in strict accordance with good animal practice as defined by UK Home Office Animal Welfare Legislation, and all animal work was approved by the Institutional Research Ethics Committee (University College London, UK) and performed under project licence number 70/7024. Pathogen free WIP-/- mice from SV129/C57/BL6 mouse strain and SV129/C57/BL6 control (wild-type) mice were bred in the facilities of Centro de Biología Molecular “Severo Ochoa” (CSIC-UAM), Madrid (Spain). These mice were housed till 6–8 weeks old in a pathogen-free animal facility. Handling of mice and all manipulations were carried out in accordance with national and European Community guidelines, and were reviewed and approved by the institutional committee for animal welfare.

### 2.2 Cell culture

DCs were generated from mouse spleens and they were infected using lentiviral vectors as previously described (Chou et al., 2006). Briefly, spleens from 6- to 8-week-old SV129 mice were homogenised through a cell strainer to obtain a cell suspension. Cells were washed twice with RPMI (Sigma, UK) containing 1% heat-inactivated foetal bovine serum (FBS) and then resuspended in RPMI supplemented with 10% FBS, 1 mM pyruvate (Sigma, UK), 1× non-essential amino acids (Sigma, UK), 2 mM glutamine (Sigma, UK), 50 µM 2-ME (Gibco BRL), 20 ng/ml recombinant mouse GM-CSF (R&D Systems) and 1 ng/ml recombinant human TGF-ß (R&D Systems) and plated at a density of 2 × 10^6^ cells/ml in 75 cm^2^ culture flasks at 37 °C in a 5% CO_2_ atmosphere. After 5 days of culture, 5 ml fresh medium were added per flask and at day 8, the cells in suspension were collected, replated and kept in suspension in fresh medium. After a total of 17–18 days ex vivo, 80–90% of the cells in culture were DCs as determined by the expression of CD11c and DEC205 by FACS analysis. Cell viability before experimental assays was tested by Trypan Blue exclusion. The mouse microvascular endothelial immortalised cell line, SVEC 4-10 (O’Connell and Edidin, 1990) was obtained from the American Type Culture collection and cells were cultured using DMEM (Sigma, UK) supplemented with 10% FBS at 37 °C in a 5% CO_2_ atmosphere.

### 2.3 Infections of DCs with lentiviral vectors

Lentiviral vector stocks were produced in 293 T cells by cotransfecting the transfer vector SFFVeGFP-WASP, the envelope plasmid pMD.G, and the packaging plasmid pCMVR8.91. 3 × 10^7^ cells were seeded onto 150 cm^2^ flasks and transfected with 10 µg DNA envelope, 30 µg DNA packaging, and 40 µg DNA transfer vector by precomplexing with 0.125 µM PEI (22 kDa) for 15 min at room temperature in Optimem. After 4 h at 37 °C, the medium was replaced with fresh DMEM 10% FCS and virus were harvested 48 and 72 h post-transfection. After filtering through a 0.45 µm-pore-size filter, the virus suspension was concentrated by centrifugation at 50,000 × *g* for 2 h at 4°C. The resulting pellet was resuspended in RPMI (Sigma) and stored at -80 °C until use. The desired number of DCs were plated on fibronectin coated coverslips (10 µg/ml) overnight in complete culture medium and then, lentivirus containing supernatant was added to the cells at an MOI between 100 and 150 and incubated for 24 h. Media was replaced for complete DC culture medium after 24 h, and cells were cultured for another 48 h to allow maximal expression of lentiviral vectors before being used in experiments.

### 2.4 Interference reflection microscopy (IRM) and analysis of adhesion turnover

DCs were plated on fibronectin (Sigma, UK) coated glass coverslips (10 µg/ml) in complete culture medium and incubated overnight at 37 °C in a 5% CO_2_ atmosphere as previously described. Coverslips were mounted onto viewing chambers in culture medium. As previously described (Chou et al., 2006; Holt et al., 2008), interference reflection micrographs were collected using a Zeiss Standard 18 microscope fitted with an incident light fluorescence attachment. Exciter and barrier filters were removed from the LP420 reflector and replaced with a narrow band-pass filter to isolate the 546 nm line of the mercury arc source. Coverslips with attached cells were observed using a Zeiss 63_Neofluar Antiflex oil-immersion objective, NA 1.25. Images were collected digitally using in-house software and processed using Adobe Photoshop^®^ version CS3 to threshold the adhesion sites of the cells with the substratum. To analyse the persistence of adhesion sites, 5 IRM images taken 30 s apart were used. Each image was thresholded to produce white adhesions on black background and then inverted as black adhesions on white background. Next, the black value of each image was divided by 5 to obtain dark grey corresponding to adhesions (i.e., 256/5 on the scale of 1–256). The images were then overlapped using the *difference* function in Adobe Photoshop. We thus obtained a composite image with 5 relevant grey levels. The lightest grey level represented pixels that were present in one of the five images (adhesion points last for 30 s), and the darkest grey level represented pixels that were present in 5 out of 5 images (i.e., adhesion points last for 150 s). Therefore, the areas of lighter grey colour pixels represent dynamic adhesions whereas areas of dark grey and black colour pixels represent increasingly stable adhesions during the selected time course of measurement. Using Mathematica™ 5.2 notebooks, we could quantify the percentage of pixels corresponding to each grey level per image, which allowed us to calculate a turnover index by dividing the percentage of pixels present in 1 or 2 frames by the percentage of pixels present in 4 or 5 frames (Holt et al., 2008). Thus, a ratio of unstable adhesion over stable adhesion in each live cell was obtained. The higher value of the turnover index represents the more dynamic of the cell adhesion. Unpaired Student's *t*-test was used to assess the significance of experimental results.

### 2.5 Immunofluorescence microscopy and quantification of size of podosome cores

Freshly prepared 10 µg/ml bovine fibronectin (Sigma) solution was incubated over sterile glass coverslips for 1 h at room temperature before plating cells. 10^5^ DCs plated on substratum-coated coverslips overnight were fixed with 4% paraformaldehyde/3% sucrose for 25 min and permeabilised with 0.05% Triton-X-100/phosphate buffered saline (PBS) for 10 min. For immunostaining against ILK, DCs were fixed with 100% ice-cold methanol for 5 min. Localisation of proteins was achieved by means of an appropriate concentration of antibody diluted in 2.5% BSA/PBS at room temperature for 1 h. After three PBS washes, samples were incubated with appropriate secondary antibodies diluted in 2.5% BSA/PBS. For localisation of filamentous actin, cells were incubated with 0.1 µg/ml Alexa 568-phalloidin (Molecular Probes) for 45 min at room temperature. Coverslips were then washed three times with PBS and twice with distilled H_2_O before being mounted in Vectashield mounting medium (Vector Laboratories, UK). Confocal images were obtained with a Zeiss LSM 510 Meta confocal laser scanning head attached to a Zeiss META Axioplan 2 microscope. LSM 510 software was used to collect sequential images from optical sections taken 0.2 µm apart along the height of podosomes in the *z* axis (the height of podosomes ranged between 0.5 and a maximum height of 1.5 µm observed in WT DCs). The same software was used to obtain merged confocal projections along the *z* axis via maxima fluorescence values. Images were exported from Database Files.mdb to TIFF files by Zeiss LSM Image Browser and processed with Adobe Photoshop 7.0 software. Projected images were also used for quantification of the area of the projection of the podosomal F-actin core and the mean fluorescence intensity of proteins surrounding the cores of podosomes at the leading edge using Zeiss LSM Image Browser.

### 2.6 Western blot

DC lysates were obtained by adding Laemmli sample buffer to plated cells. Approximately 20 µg of total cell lysate protein was loaded per lane in a 12% SDS–PAGE gel and subjected to electrophoresis. Proteins were blotted onto nitrocellulose membranes with a Bio-Rad Mini protein II transfer apparatus. Blots were blocked with 5% dried milk/TBS-T for 1 h at room temperature, incubated with indicated antibodies at 4 °C overnight. After three washes with TBS-T, immunoprobed proteins were detected by incubation with horseradish peroxidase-conjugated secondary antibodies at room temperature for 1 h. After further washes in TBS-T, immunoprobed proteins were visualised by ECL chemiluminescence kit (Amersham, UK), exposed on Hyperfilm ECL (Amersham, UK), and developed with an Imaging Systems Xograph compact X4 developer. Blots were reprobed after treating with stripping buffer at 50 °C for 30 min three times.

### 2.7 Cell transmigration assay

A confluent monolayer of SVEC 4–10 cells was generated by plating 3 × 10^4^ cells on 10 µg/ml fibronectin-coated 13-mm-diameter coverslips in 24-well plates overnight. SVEC 4-10 cells were activated to induce maximal expression of cell adhesion molecules by incubation with 100 nM LPS (Sigma) for 6 h. DCs were fluorescently labelled by incubation in CFSE (Molecular Probes, UK) and 25 × 10^3^ cells seeded per well in 0.5 ml RPMI. After 1 h, co-cultures of DCs and SVEC were washed once with PBS at 37 °C and fixed for 20 min in 4% (w/v) paraformaldehyde/3% (w/v) sucrose in PBS at 37 °C. Coverslips were stained with Alexa Fluor 568 phalloidin to detect F-actin and mounted onto slides. Three sequential confocal optical sections were taken at the top, centre and bottom of the SVEC monolayer of randomly chosen fields. We scored the percentage of DCs per coverslip found on the surface of the monolayer (apical end of the monolayer), spanning the monolayer (transmigrating), or having fully crossed the monolayer (basal end of the monolayer) for 50 DCs chosen at random per coverslip in four coverslips per experiment.

### 2.8 Matrix degradation assay

Gelatin (Sigma) was labelled with rhodamine B isothiocyanate (Sigma) by dissolving the gelatin in sodium borohydrate buffer (50 mM Na_2_B_4_O_7_ (Aldrich), 61 mM NaCl, pH 9.3) for 1 h at 37 °C and incubation with rhodamine (36 µg/ml) for 2 h at room temperature in the darkness. The buffer was changed to PBS by extensive dialysis at 4 °C over 2 days, followed by a quick spin to remove insoluble material. Sucrose was added to the sample to a final concentration of 2.5% gelatin/2.5% sucrose, aliquoted and stored for up to 21 days at 4 °C. To coat coverslips, gelatin/sucrose in PBS was warmed to 37 °C and added as a fine film, followed by crosslinking with 0.5% glutaraldehyde in PBS. Coverslips were washed 3× with PBS, and then incubated with 5 mg/ml sodium borohydride (Aldrich) in PBS for 3 min. Coverslips were then washed gently 3× in PBS and sterilised in 70% ethanol for 5 min, dried and quenched in RPMI for 1 h at 37 °C. For matrix degradation assays, 5 × 10^4^ cells were resuspended in 1 ml of DC medium, and seeded onto gelatin or fibronectin-coated coverslips in 24-well plate and incubate overnight at 37 °C. The cells were fixed in 4% paraformaldehyde in PBS, permeabilised with 0.05% Triton X-100 in PBS and blocked with 3% bovine serum albumin in PBS and incubated with appropriate primary and secondary antibodies or fluorescent phalloidin. Coverslips were mounted onto slides using Vectashield mounting medium (Vector Laboratories, UK) and visualised as described above.

### 2.9 Transwell matrigel invasion assay

The assays were carried out by using 8-µm pore filter (Transwell, 24-well plate; Corning Costar, Lowell, MA, USA) for indicated conditions. Filters were coated with 75 µl of Matrigel (Corning Matrigel^®^ Matrix, Corning, USA). The lower chambers of the transwells were filled with 700 µl of RPMI with or without murine SDF-1a (100 ng/ml). 5 × 10^5^ WT or ILK cKD dendritic cells were loaded in 500 µl of RPMI in the upper chamber of the filter. After 6 h of incubation, the upper chamber was carefully removed, and cells that had migrated to the bottom chamber were re-suspended and manually counted using a haemocytometer. The average number of migrated cells was calculated in four filters per condition.

### 2.10 Statistics

Statistical significance was assessed using Sigma Plot software. When all the samples in a given experiment followed a normal distribution we applied Student's *t*-test. For comparison of non-parametric samples we used Mann–Whitney–Wilcoxon test.

## 3 Results and discussion

### 3.1 ILK regulates podosome maturation and stability in DCs

**Fig. 1:**
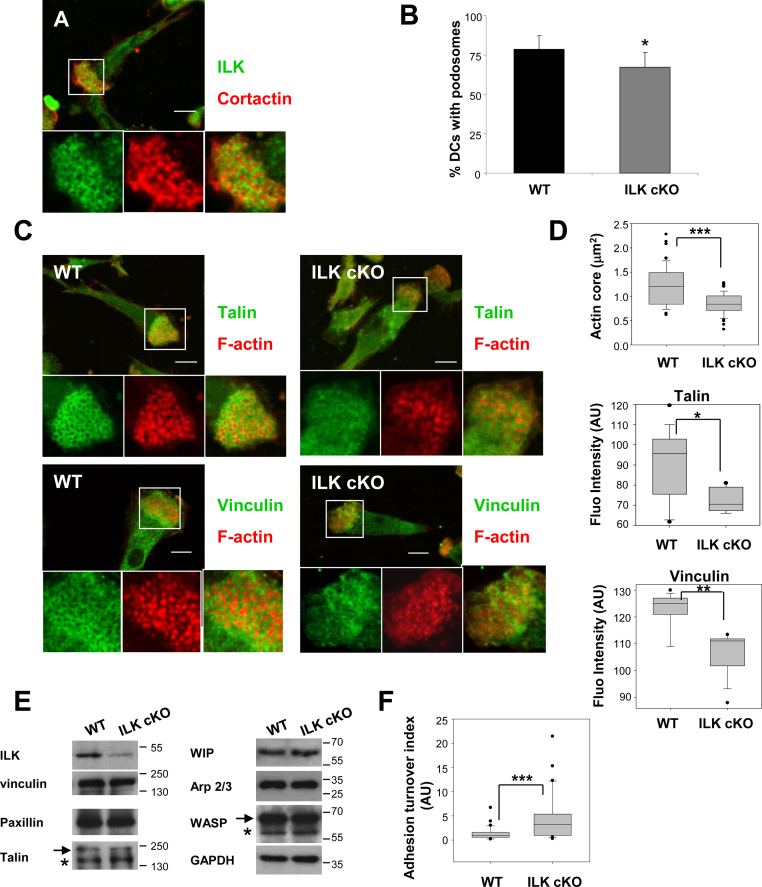
ILK regulates the accumulation of talin and vinculin in podosome rings required for podosome maturation and stabilisation. (A) Confocal micrograph showing the distribution of ILK (green) in the podosome ring and cortactin (red) in the podosome core of DCs plated on fibronectin-coated coverslips in RPMI supplemented with 10% FCS overnight and fixed with 100% ice-cold methanol. Magnifications of the boxed area with ILK and cortactin staining are shown at the bottom. Bar: 10 µm. (B) Histograms indicate the mean and SD values of the percentage of control wild type (WT) and ILK cKO DCs of 3 experiments performed with 2 mice at the time (*n* > 50 cells per mouse). Unpaired Student's *t* test was used to assess the significance of the difference between cell types, *p < 0.05. (C) Confocal micrographs showing the distribution of actin in red and talin (top panels) and vinculin (bottom panels) in green in WT and ILK cKO DCs. The images show the DC distribution of the proteins positioned ventrally in close contact with the substratum (maximum of 1.5 µm in the *z* axis from the attachment to the substratum). Magnifications of the boxed area with talin/vinculin and actin staining are shown at the bottom. Bar: 10 µm. (D) Box and whiskers diagrams showing the smallest value, the lower quartile, the median, the upper quartile and largest value of the area of the actin core and the intensities of talin and vinculin fluorescence staining in the podosome cluster in arbitrary units (AU) in WT and ILK cKO DCs. Outlying data are shown with dots. Significant differences were observed at *p < 0.05, **p < 0.01 and ***p < 0.005 as indicated (Mann–Whitney–Wilcoxon test). (E) Detection by WB of total levels of podosomal components in WT and ILK cKO DCs. GAPDH levels were used for loading control. Arrows and the asterisks mark the position of WASP and talin full length forms and the calpain-mediated cleavage products, respectively. (F) Box and whiskers diagrams showing the smallest value, the lower quartile, the median, the upper quartile and largest value of the adhesion turn over index of WT and ILK cKO DCs. ILK cKO DCs form unstable podosome-mediated adhesions as determined by IRM. (For interpretation of the references to color in this figure legend, the reader is referred to the web version of the article.)

We found that ILK was recruited to rings around podosomal cores in immature DCs (Fig. 1A). DCs derived from an ILK conditional KO mice (ILK cKO) (Herranz et al., 2012) still polarised (form distinctive protrusive and contractile poles that allow DC orientation for migration) and cells consistently showed a very small but significant reduction in the percentage of DCs containing podosomes (WT: 78.6 ± 8.5% vs ILK cKO: 67.1 ± 9.3%, Fig. 1B). The podosomes assembled by ILK deficient DCs seeded on fibronectin were distinctively less robust with F-actin cores of smaller radius and podosomal rings displaying a lower content of the integrin associated proteins talin and vinculin in comparison to DCs derived from control mice (Fig. 1C and D). This abnormal configuration of podosomal proteins did not correlate with changes in the expression of total protein (Fig. 1E). Taking together our results suggested that although ILK cKO DCs can initiate the polymerisation of actin for the assembly of the core of actin filaments, they failed to mature podosomes with a complete ring of integrin-associated proteins. We hypothesised that this morphology may correlate with higher podosome instability due to premature podosome disassembly. To test this possibility we analysed podosome turnover using IRM. The defective organisation of podosomes in ILK cKO DCs correlated with increased rate of adhesion turnover on fibronectin (Fig. 1F and Supplementary videos 1 and 2). Taken together our data indicate that ILK is not required for actin polymerisation organised in puncta during podosome initiation or DC polarity. However, ILK is essential to promote further clustering of integrin associated proteins around nascent actin cores in response to integrin ligands required for stabilisation of podosomes.

### 3.2 ILK bridges PI3K-induced podosome initiation with podosome maturation

**Fig. 2:**
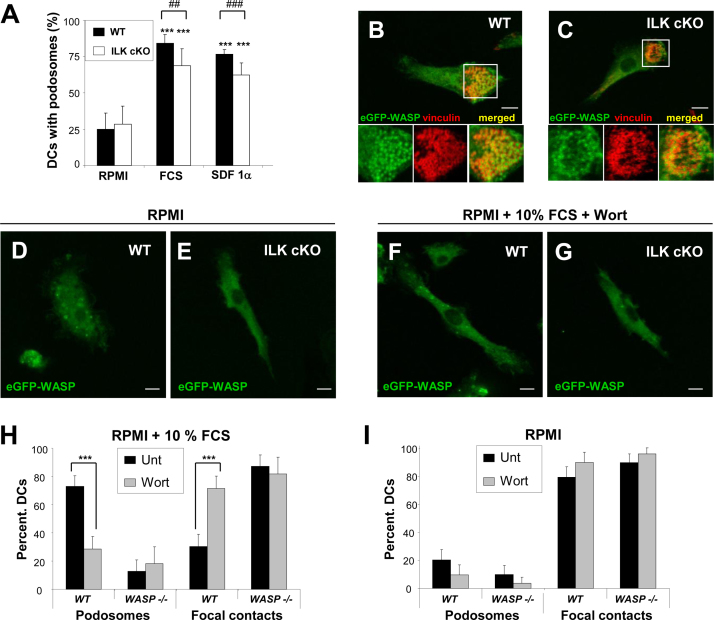
Podosomes in ILK cKO DCs follow the same WASP-dependent dynamics of initiation as in wild type (WT) DCs. (A) Histograms show the mean and SD of the percentage of DCs with podosomes that were seeded for 3 h on fibronectin-coated coverslips in RPMI only (RPMI) or supplemented with 10% FCS (FCS) or 100 ng/ml SDF1a. Unpaired Student's *t* test was used to assess the significance of the difference between DCs supplemented with FCS or SDF1a and non-stimulated DCs (RPMI) in WT or ILK cKO DCs, ***p < 0.005 or the significance of the difference between WT and ILK cKO DCs under non-stimulated (RPMI) or FCS or SDF1a stimulation, ^##^*p* < 0.01 and ^###^*p* < 0.005. (B and C) ILK cKO DCs recruit WASP to the core of podosomes similarly to WT DCs. Micrographs show the distribution of eGFP-WASP (green) and vinculin (red) in podosomes of WT (B) and ILK cKO DCs (C) stimulated with FCS to induce podosome formation. Magnifications of the boxed area with eGFP-WASP, vinculin staining and merged images are shown at the bottom. Bar: 10 µm. (D–G) Confocal micrographs showing the distribution of eGFP-WASP (green) in WT (D and F) and ILK cKO (E and G) DCs seeded on fibronectin-coated coverslip in RPMI alone left untreated (D and E) or seeded on fibronectin-coated coverslip in RPMI supplemented with 10% FCS and treated with 10 nM wortmanin for 4 h (F and G). Bar: 10 µm. (H and I) Histograms show the percentage of WT or WASP-/- DCs with podosomes or focal contacts seeded on fibronectin stimulated with FCS (H) or non-stimulated (I) that were left untreated or treated with 10 nM wortmanin for 3 h. Statistical difference between untreated and wortmanin treated cells were determined using Student's *t* test, ***p < 0.005. (For interpretation of the references to color in this figure legend, the reader is referred to the web version of the article.)

Stimulation of control and ILK cKO DCs with the chemokine SDF1a induced DC polarity and formation of podosomes in a manner indistinguishable from stimulation with serum (Fig. 2A). These results further indicate that ILK is not required for initiation of the F-actin and integrin associated protein assembly during acquisition of polarity and podosome initiation in response to soluble factors that can work as chemotactic cues for immature DCs. Clustering of WASP in discrete dot-like domains at the cell membrane is critical for the localised F-actin polymerisation that precedes organisation of circular arrays of integrins and associated proteins during podosome initiation (Chou et al., 2006; Luxenburg et al., 2012). We then aim to test whether ILK was required for the recruitment of WASP at sites of podosome initiation. WASP clustering in podosome cores was still observed in stimulated ILK cKO DCs (Fig. 2B and C). In the absence of soluble factors (cells cultured in RPMI only), WASP appeared homogenously distributed in the cytoplasm in both control and ILK cKO DCs (Fig. 2D and E) correlating with impaired podosome formation (Fig. 2A) and DC adhesion through focal contacts instead. These results indicate that in the absence of ILK, WASP-mediated signalling required for actin polymerisation during podosome initiation remains unaffected. We further investigated whether WASP activation leading to formation of podosome cores is independent of ILK.

WASP acquires an active open conformation in the podosome core in macrophages (Dovas et al., 2009) inducible by PI3K activity upon CSF-1 stimulation (Cammer et al., 2009). Inhibition of PI3K activity with wortmanin in control or ILK cKO DCs in the presence of serum prevented the organisation of WASP into dot-shaped clusters and instead it distributed homogenously in the cytosol (Fig. 2F and G). Taken together our results indicate that the initial recruitment of WASP to cores during podosome formation in response to soluble factors is independent of ILK action but requires PI3K activity. These data also suggest that focal contacts form independently of PI3K activity in non-polarised DCs.

To test this possibility, we investigated whether focal contacts organised independently of PI3K activity in WT DCs, in WASP-/- and WIP-/- DCs. We have previously reported that both WASP-/- and WIP-/- DCs fail to assemble podosomes and instead attach to integrin ligands using focal contacts (Calle et al., 2008; Chou et al., 2006; Monypenny et al., 2011). Treatment of WT DCs seeded in the presence of serum with the PI3K inhibitor wortmanin resulted in podosome disassembly and adhesion through focal contacts (Fig. 2H), correlating with the observed homogenous distribution of WASP in the cytosol (Fig. 2F). WASP-/- DCs treated with wortmanin remained attached on fibronectin using focal contacts (Fig. 2H). Additionally, formation of focal contacts by WT or WASP-/- DCs in the absence of serum stimulation was not inhibited by treatment with wortmanin (Fig. 2I). Similar results were also obtained when WIP-/- DCs were treated with wortmanin (Supplementary Fig. I) and when DCs were treated with a second PI3K inhibitor LY294002 (data not shown). Taken together, our results indicate that in the absence of stimulation for podosome formation, DCs can still attach on integrin ligands using focal contacts that form independently of PI3K-mediated WASP activity.

Overall, our results show that WASP and WIP but not ILK are key regulators for PI3K-dependent F-actin and integrin remodelling for DC polarisation and podosome initiation. ILK is critical for the accumulation of integrins forming a ring around the nascent actin podosome cores leading to podosome maturation and stability.

### 3.3 ILK is required for podosome-mediated ECM degradation and tissue invasion across ECM barriers

**Fig. 3:**
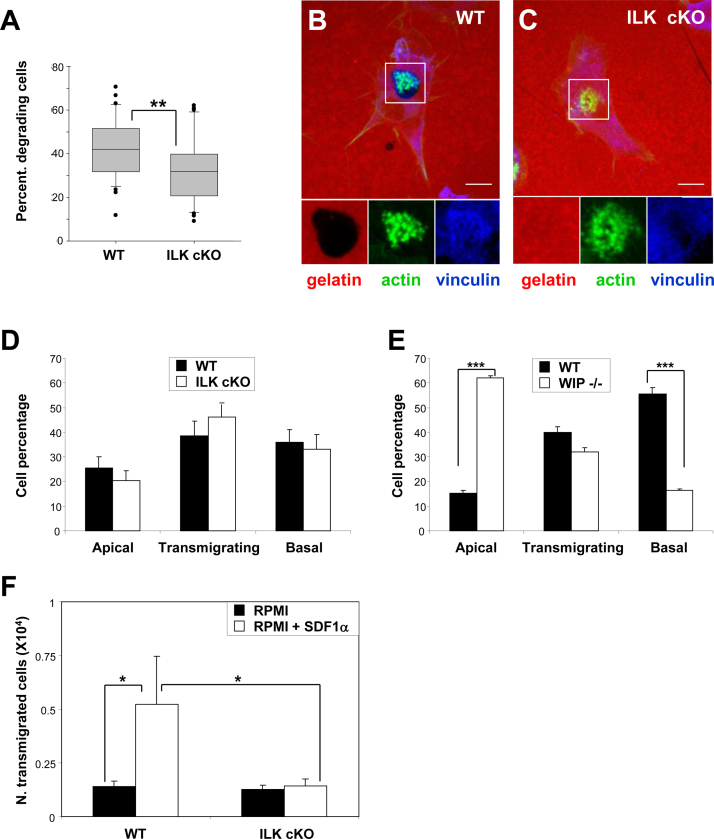
Reduced matrix degradation and impaired invasive migration of ILK cKO DCs across Matrigel. (A) Box and whiskers diagrams showing the percentage of DCs with an associated subjacent area of gelatin degradation per field of view. **p < 0.01, Mann–Whitney–Wilcoxon test. (B and C) Confocal micrographs showing the distribution of TRITC-gelatin (red) actin (green) and vinculin (blue) in WT (B) and ILK cKO (C) DCs seeded on TRITC-gelatin overnight. Magnifications of the boxed area with TRITC-gelatin, actin and vinculin staining are shown at the bottom. ILK cKO DCs failed to mature podosomes correlating with low degradation of subjacent gelatin. Bar: 10 µm. (D and E) Histograms indicate the average percentage and SE of DC transmigrated, transmigrating or retained on the apical surface of a monolayer of LPS-activated endothelial cells after 1 h of co-culture. Data were obtained from experiments comparing the transendothelial migration between control (WT) and ILK cKO DCs (D) and WT and WIP-/- DCs (E). (F) Histograms show the average of the number cells (×10^4^) that transmigrated across a Matrigel coated filters towards SDF1a (100 ng/ml) to test the invasive capacity of DCs. Statistical differences between were determined using Student's *t* test, *p < 0.05, ***p < 0.005. (For interpretation of the references to color in this figure legend, the reader is referred to the web version of the article.)

Podosomes are sites for degradation of the extracellular matrix by DCs (Banon-Rodriguez et al., 2011; Linder, 2009) and they have been shown to play a role in degradation of the basement membrane in other cell types during tissue invasion and remodelling (Schoumacher et al., 2011). We found that the impaired maturation and stability of podosomes in ILK cKO DCs (Fig. 1) correlated with a decrease in the degradation of gelatin (Fig. 3A–C).

Recruitment of circulating immature DCs to sites of inflammation requires extravasation across the endothelial barrier, a process that is regulated by WASP (Calle et al., 2006b; Macpherson et al., 2012) followed by degradation of the basal membrane to penetrate the subjacent tissue. Maintenance of polarity in ILK deficient DCs correlated with efficient transendothelial migration (Fig. 3D), whereas lack of WIP (which regulates DC polarity and podosome initiation (Banon-Rodriguez et al., 2011; Chou et al., 2006)) inhibited transendothelial migration of DCs (Fig. 3E) similar to the case seen with lack of WASP (Macpherson et al., 2012). Our results suggest that regulation of WASP/WIP-dependent DC polarity is crucial for DC transmigration across the endothelium. However, ILK-mediated podosome stability in response to integrin ligands does not appear to be essential for transendothelial migration of DCs as cells can still polarise and acquire a motile phenotype. We have previously reported that in transmigrating DCs, podosomes assemble on the basal matrix contacting surface of DCs located beneath endothelial monolayers (Calle et al., 2008). Our current results show that ILK is required for stabilisation and degradative activity of podosomes, suggesting that ILK may be involved in the migration of DCs across ECM barriers. We then tested the invasive capacity of WT and ILK cKO DCs across Matrigel gels. In comparison to WT DCs, ILK cKO DCs failed to effectively cross the Matrigel gel and mobilise towards a gradient of SDF1a (Fig. 3F).

ILK was initially identified as a potential Ser/Thr kinase. However, the significance of the kinase activity of ILK in vivo remains largely controversial (Hannigan et al., 2011; Wickstrom et al., 2010). ILK is thought to function primarily as an adaptor protein that plays a key role in outside-in signalling by reinforcing the link between integrin tails and F-actin (Bottcher et al., 2009), promoting maturation of focal adhesions (Stanchi et al., 2009). Overall, our work indicates that ILK is required for the accumulation of integrins around the nascent cores of F-actin during maturation of podosomes in DCs. We also show that this ILK-mediated process is crucial for degradation of the ECM. Podosomes belong to a larger family of adhesions involved in tissue invasion called invadosomes, which includes invadopodia (Linder, 2009). Similarly to our findings in podosomes, ILK also regulates maturation as well as matrix degradation mediated by invadopodia in cancer cells (Branch et al., 2012). Our results suggest a possible role of ILK in maturation of podosomes for degradation of the endothelial basement membrane by immature DCs for tissue invasion after transmigration across the endothelium. These findings support the previously proposed role of leucocyte podosomes for degradation of the endothelial basement membrane (Wolosewick, 1984).

## 4 Conclusions

In summary, we have found that ILK is not required for DC polarity or podosome initiation, which is instead regulated by PI3K-dependent spatial organisation of WASP, leading to localised actin polymerisation and adhesion remodelling. Our work shows that ILK bridges PI3K-induced DC polarisation for migration and podosome initiation with podosome maturation and stabilisation in response to integrin ligands. We conclude that ILK is essential for the accumulation of integrin associated proteins in the ring surrounding nascent podosome actin cores leading to podosome maturation and functionality to degrade the extracellular matrix.

## Appendix A Supplementary data

Supplementary Video 1 Live IRM microscopy imaging of WT DCs seeded on fibronectin-coated coverslips overnight in RPMI supplemented with 10% FCS and assembled in viewing chambers. Micrographs were taken 10 s apart and displayed at 10 frames per second.

Supplementary Video 2 Live IRM microscopy imaging of ILK cKO DCs seeded on fibronectin-coated coverslips overnight in RPMI supplemented with 10% FCS and assembled in viewing chambers. Micrographs were taken 10 s apart and displayed at 10 frames per second.

Supplementary Fig. I Focal contacts in WIP-/- DCs form independently of PI3K activity. Histograms show the percentage of WT or WIP-/- DCs with podosomes or focal contacts seeded on fibronectin stimulated with 10% FCS (A) or non-stimulated (B) that were left untreated or treated with 10 nM wortmanin for 3 h. Statistical difference between untreated and wortmanin treated cells were determined using Student's *t* test, ****p* < 0.005.
