# Supplementary figures and images for: Integrin linked kinase (ILK) regulates podosome maturation and stability in dendritic cells
Source: Int J Biochem Cell Biol. 2014 May;50(100):47–54. doi: 10.1016/j.biocel.2014.01.021 (PMC3998073; doi:10.1016/j.biocel.2014.01.021)

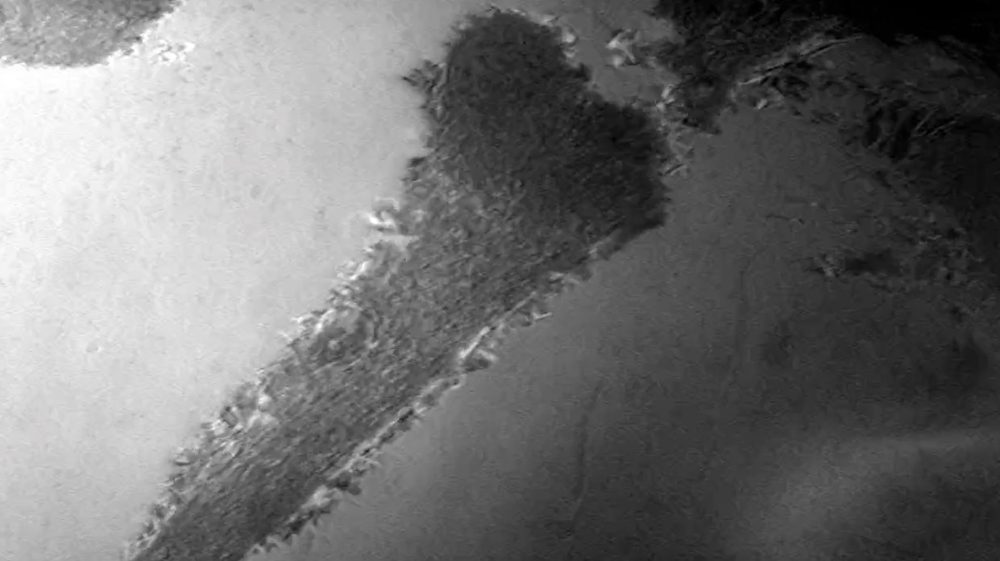

Supplement: Supplementary Video 1 — Live IRM microscopy imaging of WT DCs seeded on fibronectin-coated coverslips overnight in RPMI supplemented with 10% FCS and assembled in viewing chambers. Micrographs were taken 10 s apart and displayed at 10 frames per second. [file mmc1.jpg]

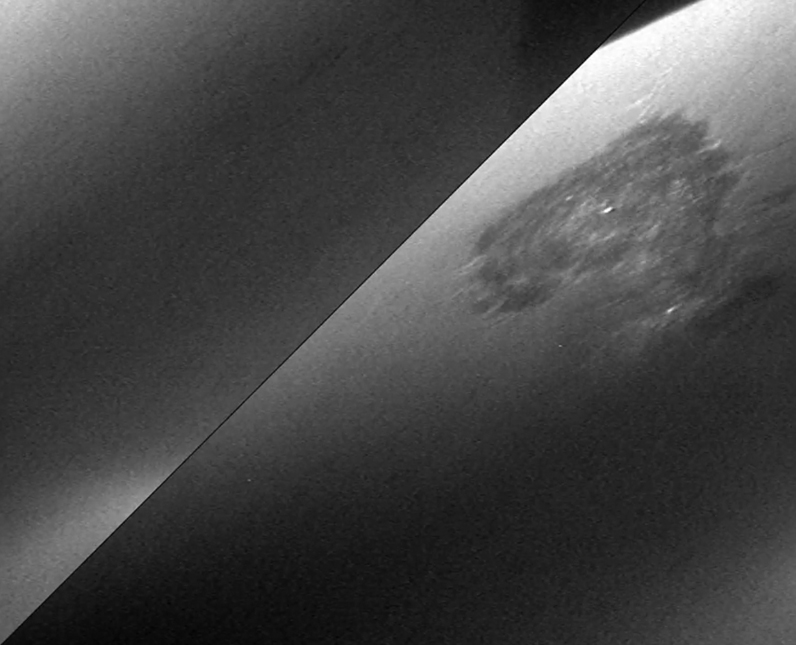

Supplement: Supplementary Video 2 — Live IRM microscopy imaging of ILK cKO DCs seeded on fibronectin-coated coverslips overnight in RPMI supplemented with 10% FCS and assembled in viewing chambers. Micrographs were taken 10 s apart and displayed at 10 frames per second. [file mmc2.jpg]
