# Supplementary material for: Integrin linked kinase (ILK) regulates podosome maturation and stability in dendritic cells
Source: Int J Biochem Cell Biol. 2014 May;50(100):47–54. doi: 10.1016/j.biocel.2014.01.021 (PMC3998073; doi:10.1016/j.biocel.2014.01.021)
Supplement: Supplementary Fig. I — Focal contacts in WIP-/- DCs form independently of PI3K activity. Histograms show the percentage of WT or WIP-/- DCs with podosomes or focal contacts seeded on fibronectin stimulated with 10% FCS (A) or non-stimulated (B) that were left untreated or treated with 10 nM wortmanin for 3 h. Statistical difference between untreated and wortmanin treated cells were determined using Student's t test, ***p < 0.005. [file mmc3.ppt]

## Slide 1
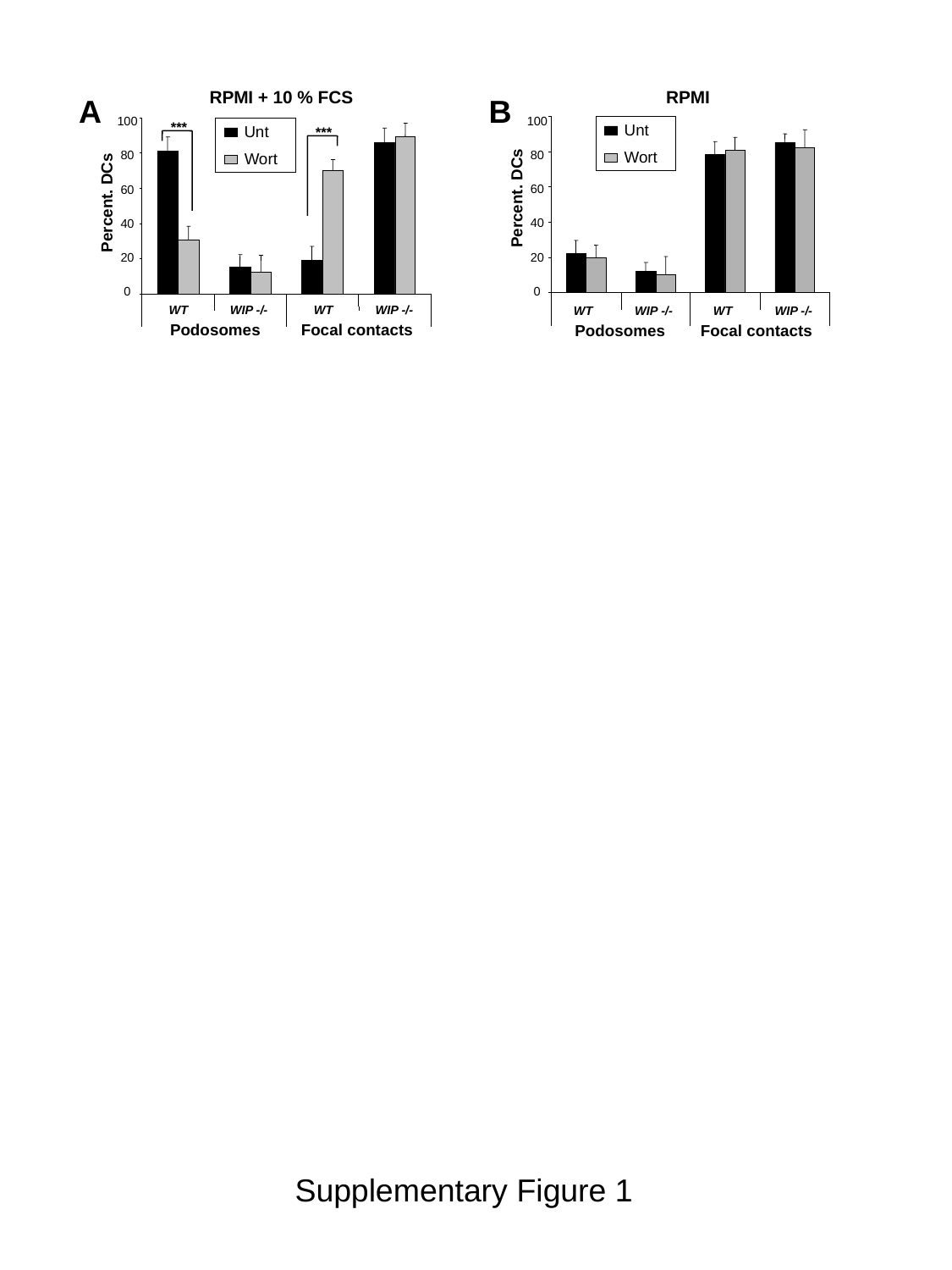

A
B
RPMI + 10 % FCS
RPMI
***
100
100
Unt
Wort
***
Unt
80
80
Wort
60
60
 Percent. DCs
Percent. DCs
40
40
20
20
0
0
WT
WIP -/-
WT
WIP -/-
WT
WIP -/-
WT
WIP -/-
Podosomes
Focal contacts
Podosomes
Focal contacts
Supplementary Figure 1
